# Total DNA methylation as a biomarker of DNA damage and tumor malignancy in intracranial meningiomas

**DOI:** 10.1186/s12885-020-06982-3

**Published:** 2020-06-03

**Authors:** Anna-Maria Barciszewska

**Affiliations:** 1 Intraoperative Imaging Unit, Chair and Department of Neurosurgery and Neurotraumatology, Karol Marcinkowski University of Medical Sciences, Przybyszewskiego 49, 60-355 Poznan, Poland; 2 Department of Neurosurgery and Neurotraumatology, Heliodor Swiecicki Clinical Hospital, Przybyszewskiego 49, 60-355 Poznan, Poland

**Keywords:** Meningioma, DNA methylation, 5-methylcytosine, Biomarker, DNA damage

## Abstract

**Background:**

Meningiomas are the most common primary intracranial tumors in adults. They are initially detected with neuroimaging techniques, but definite histological diagnosis requires tumor surgery to collect tumor tissue. Gross total resection is an optimal and final treatment for the majority of patients, followed by radiotherapy in malignant or refractory cases. However, there are a lot of uncertainties about i.a. the need for intervention in incidental cases, estimation of growth kinetics, risk of malignant transformation, or response to radiotherapy. Therefore a new diagnostic approach is needed. It has already been shown that epigenetics plays a crucial role in cancer biology, development, and progression. DNA methylation, the presence of 5-methylcytosine in DNA, is one of the main elements of a broad epigenetic program in a eukaryotic cell, with superior regulatory significance. Therefore, we decided to look at meningioma through changes of 5-methylcytosine.

**Methods:**

We performed an analysis of the total amount of 5-methylcytosine in DNA isolated from intracranial meningioma tissues and peripheral blood samples of the same patients. The separation and identification of radioactively labeled nucleotides were performed using thin-layer chromatography.

**Results:**

We found that the 5-methylcytosine level in DNA from intracranial meningiomas is inversely proportional to the malignancy grade. The higher the tumor WHO grade is, the lower the total DNA methylation. The amount of 5-methylcytosine in tumor tissue and peripheral blood is almost identical.

**Conclusions:**

We conclude that the total DNA methylation can be a useful marker for brain meningioma detection, differentiation, and monitoring. It correlates with tumor WHO grade, and the 5-methylcytosine level in peripheral blood reflects that in tumor tissue. Therefore it’s applicable for liquid biopsy.

Our study creates a scope for further research on epigenetic mechanisms in neurooncology and can lead to the development of new diagnostic methods in clinical practice.

## Background

Meningiomas are the most frequent primary central nervous system tumors (37.6%), reaching an annual incidence of 8.56/100000 [1]. The occurrence rate for meningioma increases with age (adults age 65 years and older), is higher in females than in males (female: male ratio is 2.21), and in Blacks than in Whites [1]. They present with overall five-year survival of 68.2% for malignant and 88.0% for non-malignant cases, and a relative 10-year survival ranging from 61.7% for malignant and 83.7% for non-malignant meningiomas, respectively [1]. The well-validated risk factor for those tumors is ionizing radiation [1, 2]. The majority (80.5%) of meningiomas are low grade (I) tumors, but 17.7% are atypical (grade II), and 1.7% anaplastic (grade III) according to the WHO 2016 criteria [1, 3]. The WHO brain tumors’ classification lists 15 meningioma subtypes, most of them allotted to WHO grade I (Meningothelial, Fibrous/Fibroblastic, Transitional/Mixed, Psammomatous, Angiomatous, Microcystic, Secretory, Lymphoplasmacyte-rich, Metaplastic). Other variants, presenting with a higher likelihood of recurrence and aggressive behavior, belong either to grade II (Atypical, Clear-cell, Chordoid) or III (Anaplastic/Malignant, Rhabdoid, Papillary) [4]. Those subtypes are distinguished by their specific microscopic features. However, some genetic and clinical relevancies are also known, as the presence of *NF2* mutations (up to 80% in fibroblastic and transitional meningiomas, only in 25% of meningothelial ones, and very rarely in the secretory), prevalence in specific locations (Clear-cell meningiomas in the spinal cord and posterior fossa, Chordoid - typically supratentorial), and age groups (as Papillary type in children) [4]. Grading is entirely based on histological features and does not include molecular markers. Mitotic count of 4 or more per 10 high power fields and brain invasion are diagnostic criteria of atypical meningioma, WHO grade II. The existing classification and grading system have prognostic value. However, they possess some shortcomings, such as ill-defined subtypes’ parameters and grading criteria that are susceptible to arbitrary judgment [3].

Watchful waiting and surgery are the first-line treatments of meningiomas. Adjuvant radiotherapy and radiosurgery are taken into account for atypical cases and indicated for anaplastic meningiomas [5]. The most of meningiomas may be cured by surgical resection. However, ca. 20% of tumors present aggressive clinical behavior with recurrence or progression, which results in significant morbidity and mortality of affected patients. Skull base meningiomas are usually the most challenging cases because of vascular and nervous structures involvement in and adjacent to the tumor, as well as resistance to radiation therapy. That limits the possibility of their total surgical removal and radiotherapeutic approach. Chemotherapy has not yet been proven to be effective in meningiomas, but there are several clinical trials ongoing [6].

The vital challenge for neuropathological evaluation of meningioma is not the identification of the entity, but its subtyping and grading. However, the standard WHO scheme does not allow for sufficient prediction of the recurrence of the tumor, overall prognosis, and systemic treatment options [7]. Therefore, for completion of the histological classification with therapeutic insights, the molecular characterization of these tumors is essential. However, the pathogenic mechanisms leading to meningioma development is still undefined. In 60–80% of sporadic meningiomas, the mutation in neurofibromatosis type 2 gene (*NF2*) leading to its inactivation occurs [8, 9]. The ongoing research involves the exploration of other genetic mutations, such as *SMO*, *AKT1*, *TERT*, *BAP1*, *KLF4*, *TRAF7*, as well as the methylation profile [7, 10]. *TERT* gene alterations, including promoter mutations, gene translocations, and DNA amplifications, appear to be a biomarker significantly predicting higher recurrence and mortality rate in meningiomas [11]. An accumulation of cytogenetic aberrations, usually 1p, 10, and 14q losses, was shown to be associated with higher malignancy and increased recurrence rate [4, 12, 13]. Grade II and III meningiomas display a more complex molecular and cytogenetic background than benign (grade I) ones. That includes the inactivation of tumor suppressor genes, oncogenes’ activation, and modifications in various genes taking part in multiple cellular processes [14, 15]. However, none of those alterations appeared to have prognostic relevance and biomarker potential. Moreover, most meningiomas do not present well defined genetic abnormalities, which may imply that other mechanisms, e.g., epigenetic aberrations, may influence tumor behavior [16]. However, the meningiomas’ epigenetic landscape remains incomplete.

DNA methylation, the presence of 5-methylcytosine (m^5^C) in DNA, is one of the main elements of a broad epigenetic program in a eukaryotic cell, with the regulatory significance. It results in silencing or reactivation of cancer-related genes [17]. Distinctiveness and specificity of m^5^C as an epigenetic marker lies in high stability of C-C bond in m^5^C, as well as lack of specific demethylating enzymes. Therefore the process of methyl group removal (demethylation) is carried out through enzymatic oxidation with Ten-Eleven Translocation (TET) enzymes or through spontaneous oxidation by reactive oxygen species (ROS) action [18]. It has been shown that cancer development and progression is the result of the disruption of the redox balance of the cell, which is induced by enhanced reactive oxygen species (ROS) generation, their accumulation, and antioxidant enzymes downregulation [19]. ROS cause damage to DNA and other cell components, promote epigenetic alterations, interact with oncogenes and tumor suppressor genes, and finally modulate immunological responses [20, 21]. ROS-induced m^5^C damage leads to its demethylation and deamination [22]. It results in the global (genomic) hypomethylation of cellular DNA. Therefore, total DNA methylation (m^5^C contents) is a sensitive marker for carcinogenesis as an effect of the oxidative stress, ROS formation, and damage reactions [23]. We have recently shown that DNA methylation status (hypomethylation) reflects the level of oxidative stress in the cell [24].

The results of gene-candidate studies [25–29] and genome-wide analysis [30, 31] in meningiomas suggested that aberrant DNA promoter methylation may contribute to the initiation and progression of those tumors.

To evaluate the role of DNA methylation in the development and progression of meningiomas, we have analyzed the total DNA methylation level in tumor and peripheral blood samples from intracranial meningioma patients undergoing surgical resection. For the estimation of m^5^C contents, we used a sensitive and straightforward two-dimensional thin-layer chromatography (TLC) technique of radioactively labeled nucleotides separation ([γ^32^P] post labeling method) [32, 33]. Through a detailed analysis of the total DNA methylation in intracranial meningiomas, we propose a new epigenetic approach for meningioma characterization, that can potentially be practically applied in clinical diagnostics, and treatment monitoring.

## Methods

### Ethics approval and consent to participate

Blood and tissue molecular testing was approved by the Bioethical Committee of Karol Marcinkowski University of Medical Sciences, Poznan (896/9; 838/12). All participants provided informed and written consent to donate their peripheral blood and tumor tissue samples for research.

### Collection of tumor and peripheral blood samples

The brain meningioma tissue samples were collected from 100 consecutive patients who underwent brain tumor surgery at the Department of Neurosurgery and Neurotraumatology of the Karol Marcinkowski University of Medical Sciences in Poznan between 2004 and 2012. In 29 of those patients, peripheral blood samples were also collected preoperatively. The peripheral blood samples were also taken from 30 generally healthy individuals (not related to the cases, volunteers, with no known pathologies, without regular drug intake, non-smokers) comprising the control group. Tumor and peripheral blood samples were immediately frozen, then stored at − 80 °C.

Demographic and clinical data were extracted from the patient’s medical records. Intracranial tumor samples were routinely neuropathologically evaluated to determine their histological types and grades.

### DNA isolation from tumor tissue samples

The extraction of genomic DNA from tumor tissue samples was performed with a commercially available kit (A&A Biotechnology). The samples were first incubated with proteinase K, then with RNaseA. The supernatant, obtained after centrifugation, was applied to a mini-column. DNA elution was done with Tris-buffer pH 8.5. It was then stored at − 20 °C for further analysis. DNA UV absorbance was measured at 260 and 280 nm for checking DNA purity. The A_260_/A_280_ ratio was 2.0–2.1.

### DNA isolation from peripheral blood samples

DNA from a peripheral blood sample (7.5 ml) was isolated by lysis with 30 ml of cold (4 °C) buffer of NH_4_Cl (155 mM), KHCO_3_ (10 mM), and Na_2_EDTA (0.1 mM), in pH 7.4 for 30 min., then centrifuged at 3000 rpm for 10 min. in 4 °C. The pellets were resuspended in 10 ml of the above-mentioned buffer and centrifuged again. The cell lysate was resuspended in 5 ml of buffer containing NaCl (75 mM), Na_2_EDTA (1 mM), in pH 8.0, and digested with protease K solution (25 μl, concentration 10 μg/μl), and 20% SDS (250 μl) for 16 h at 55 °C. After the incubation 5 M NaCl (1500 μl) was added, then tube shaken vigorously for 15 s, and centrifuged at 4000 rpm for 15 mins at room temperature. The DNA precipitation was done with two volumes of cold ethanol. The solution was centrifuged at 4000 rpm for 20 mins at room temperature. Ethanol was removed, and the precipitate dissolved in distilled water (100 μl). DNA UV absorbance was measured at 260 and 280 nm for checking DNA purity. The A_260_/A_280_ ratio was 2.0–2.1. The amount of DNA was calculated using the standard relation: 1 OD (absorbance at 260 nm) = 50 μg DNA. OD value was measured with the spectrophotometer.

### DNA hydrolysis, Labelling and TLC chromatography

**Fig. 1:**
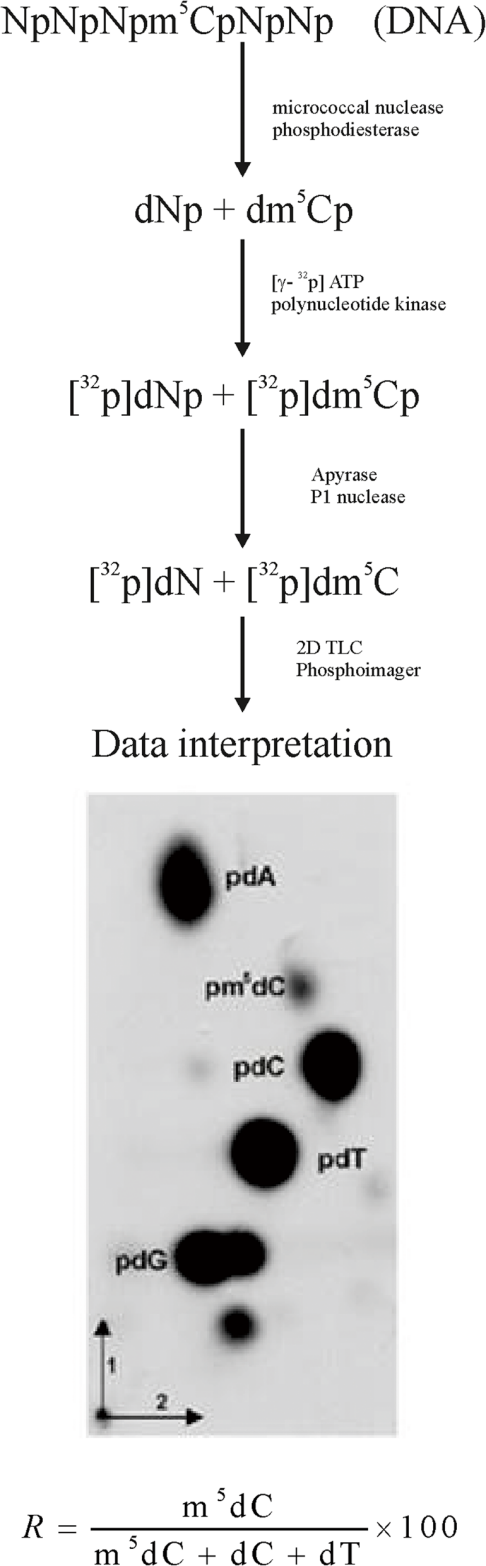
Flowchart of total genomic m^5^C estimation. Isolated DNA is hydrolyzed to 3′-mononucleotides (Np, A - adenosine, G - guanosine, C - cytidine, T - thymidine). The hydrolysate is labeled with [γ-^32^P] ATP, dephosphorylated (detachment of 3′ phosphate), and separated in two dimensions with TLC. The chromatogram is evaluated with phosphoimager, and the spots’ intensities are measured. Those values are used for the calculation of the R coefficient according to the given equation [33]

The amount of water solution containing 1 μg of DNA was placed in 1.5 ml Eppendorf tube and dehydrated in a vacuum concentrator. Dried DNA was dissolved in CaCl_2_ (10 mM) succinate buffer (pH 6.0), and then digested with the mixture of spleen phosphodiesterase II (0.001 units), and micrococcal nuclease (0.02 units) in the total volume of 3.5 μl for 5 h at 37 °C. DNA digest (0.17 μg) was labeled with [γ-^32^P] ATP (1 μCi; stock solution: 6000 Ci/mM; Hartmann Analytic GmbH), and T4 polynucleotide kinase (1.5 units) in 10 mM bicine-NaOH pH 9.7 (3 μl) buffer containing MgCl_2_ (10 mM), DTT (10 mM), and spermidine (1 mM). After 30 min. at 37 °C, apyrase (3 μl, 10 units/ml) in the same buffer was added, and the solution was incubated for another 30 min. The 3′ nucleotide phosphates were cleaved off with RNase P1 (0.2 μg) in ammonium acetate buffer (500 mM, pH 4.5). Identification of [γ^32^P]m^5^C was performed with a thin-layer chromatography (TLC) on cellulose plates (Merck, Germany) in two dimensions using solvent system: isobutyric acid: NH_4_OH:H_2_O (66:1:17 v/v) - first dimension, and 0.2 M sodium phosphate (pH 6.8)-ammonium sulfate-*n*-propyl alcohol (100 ml/60 g/1.5 ml) - second dimension. Radioactive spot detection was performed with the Phosphoimager Typhoon Screen (Pharmacia, Sweden), and ImageQuant Software (GE Healthcare, USA) was used for image analysis. The testing was repeated thrice for each probe, and the statistic software was used for results evaluation. The amount of radioactive material (spot intensity) corresponding to m^5^C, C (cytosine), and T (thymine) was used for calculations. The global DNA methylation was calculated as R = (m^5^C/(m^5^C + C + T)) × 100 (Fig. 1), because cytosine and thymine are formed in the m^5^C oxidation process [33].

### Statistical analysis

Statistical analysis (descriptive statistics, ANOVA test, correlation) was performed with STATISTICA 13.3 (Statsoft Poland) software.

## Results

### Patients’ characteristics

The analyzed cohort consisted of 100 individuals diagnosed with brain meningioma, aged from 27 to 80 years. Patients within the age range of 51–60 years (37 individuals) comprised the largest subgroup, followed by the age group of 61–70 (27 individuals). The median age of patients at the time of tumor diagnosis was 57.4 ± 10.8 years. There were 30 (30.0%) males and 70 (70.0%) females. The histological types and grades (from I – least malignant, to III most malignant), as well as numeric data from total DNA methylation analysis, are shown in Table 1.

**Table 1:** The list of 100 patients with intracranial meningioma evaluated in this study. The histological types and grades were estimated in routine pathological report

Case	Histological type	Grade	Age range	R tissue	SD R tissue	R blood	SD R blood
1.	Meningothelial meningioma	I	21–30	1.63	0.02		
2.	Meningothelial meningioma	I	21–30	1.56	0.04	1.55	0.06
3.	Meningothelial meningioma	I	41–50	1.63	0.09		
4.	Meningothelial meningioma	I	41–50	1.51	0.05		
5.	Meningothelial meningioma	I	41–50	1.65	0.03		
6.	Meningothelial meningioma	I	41–50	1.62	0.06	1.69	0.1
7.	Meningothelial meningioma	I	41–50	1.57	0.09		
8.	Meningothelial meningioma	I	41–50	1.59	0.16		
9.	Meningothelial meningioma	I	41–50	1.64	0.08	1.46	0.06
10.	Meningothelial meningioma	I	41–50	1.63	0.09		
11.	Meningothelial meningioma	I	41–50	1.59	0.08		
12.	Meningothelial meningioma (brain parenchyma infiltration)	II	41–50	1.61	0.04		
13	Meningothelial meningioma	I	51–60	1.61	0.08		
14.	Meningothelial meningioma	I	51–60	1.59	0.1	1.75	0.12
15.	Meningothelial meningioma	I	51–60	1.55	0.04		
16.	Meningothelial meningioma	I	51–60	1.55	0.09	1.60	0.12
17.	Meningothelial meningioma	I	51–60	1.57	0.07	1.52	0.09
18.	Meningothelial meningioma	I	51–60	1.51	0.1		
19.	Meningothelial meningioma	I	51–60	1.71	0.01		
20.	Meningothelial meningioma	I	51–60	1.59	0.11		
21.	Meningothelial meningioma	I	51–60	1.56	0.01		
22.	Meningothelial meningioma	I	51–60	1.48	0.05		
23.	Meningothelial meningioma (brain parenchyma infiltration)	II	51–60	1.13	0.04	1.48	0.08
24.	Meningothelial meningioma	I	51–60	1.61	0.03	1.51	0.09
25.	Meningothelial meningioma	I	51–60	1.56	0.03		
26.	Meningothelial meningioma	I	51–60	1.60	0.04		
27.	Meningothelial meningioma	I	51–60	1.58	0.09		
28.	Meningothelial meningioma	I	61–70	1.53	0.07		
29.	Meningothelial meningioma	I	61–70	1.58	0.07	1.63	0.02
30	Meningothelial meningioma	I	61–70	1.55	0.05		
31.	Meningothelial meningioma	I	61–70	1.53	0.06	1.63	0.04
32.	Meningothelial meningioma	I	61–70	1.71	0.06		
33.	Meningothelial meningioma	I	61–70	1.73	0.03		
34.	Meningothelial meningioma	I	61–70	1.54	0.05		
35	Meningothelial meningioma	I	61–70	1.56	0.05	1.68	0.02
36.	Meningothelial meningioma	I	71–80	1.53	0.09		
37.	Meningothelial meningioma	I	71–80	1.57	0.07		
38.	Meningothelial meningioma	I	71–80	1.52	0.09		
39.	Meningothelial meningioma, partially cellular	I	31–40	1.54	0.03		
40.	Meningothelial meningioma, partially cellular	I	51–60	1.61	0.04		
41.	Meningothelial meningioma	I	51–60	1.72	0.07		
42.	Meningothelial meningioma	I	51–60	1.63	0.03		
43.	Angiomatous and microcystic meningioma	I	41–50	1.54	0.1		
44.	Angiomatous meningioma	I	41–50	1.52	0.09		
45.	Angiomatous meningioma	I	51–60	1.50	0.05	1.33	0.06
46.	Angiomatous meningioma	I	51–60	1.46	0.09		
47.	Angiomatous meningioma	I	51–60	1.48	0.02	1.51	0.07
48.	Angiomatous meningioma	I	61–70	1.49	0.02	1.45	0.04
49.	Angiomatous meningioma	I	61–70	1.51	0.06		
50.	Angiomatous meningioma	I	61–70	1.46	0.09		
51.	Angiomatous meningioma	I	61–70	1.51	0.07	1.64	0.08
52.	Angiomatous meningioma	I	61–70	1.54	0.06	1.59	0.1
53.	Angiomatous meningioma	I	61–70	1.48	0.05		
54.	Fibrous meningioma	I	41–50	1.54	0.02		
55.	Fibrous meningioma	I	41–50	1.57	0.12		
56.	Fibrous meningioma	I	41–50	1.58	0.11		
57.	Fibrous meningioma	I	51–60	1.58	0.08		
58.	Fibrous meningioma	I	51–60	1.56	0.06		
59.	Fibrous meningioma	I	51–60	1.59	0.06		
60.	Fibrous meningioma	I	51–60	1.54	0.06	1.51	0.06
61.	Fibrous meningioma	I	51–60	1.53	0.07		
62.	Fibrous meningioma	I	51–60	1.49	0.12		
63.	Fibrous meningioma	I	51–60	1.49	0.06		
64.	Fibrous meningioma, partially psammomatous (brain parenchyma infiltration)	II	61–70	1.41	0.06	1.57	0.04
65.	Fibrous meningioma	I	61–70	1.71	0.09	1.67	0.04
66.	Fibrous meningioma	I	61–70	1.52	0.11		
67.	Fibrous meningioma	I	61–70	1.53	0.12		
68.	Fibrous meningioma	I	61–70	1.57	0.1	1.68	0.08
69.	Fibrous meningioma	I	61–70	1.56	0.08		
70.	Fibrous meningioma	I	61–70	1.45	0.07		
71.	Fibrous meningioma	I	61–70	1.52	0.02		
72.	Fibrous meningioma	I	71–80	1.62	0.02	1.67	0.12
73.	Fibrous meningioma	I	71–80	1.55	0.03		
74.	Fibrous meningioma	I	71–80	1.54	0.07		
75.	Fibrous meningioma	I	51–60	1.61	0.09		
76.	Psammomatous meningioma	I	51–60	1.58	0.04		
77.	Psammomatous meningioma	I	51–60	1.52	0.07		
78.	Psammomatous meningioma	I	51–60	1.46	0.07		
79.	Psammomatous meningioma	I	71–80	1.59	0.05		
80.	Atypical meningioma	II	21–30	1.57	0.08		
81.	Atypical meningioma	II	51–60	1.46	0.11	1.45	0.04
82.	Atypical meningioma	II	61–70	1.53	0.09	1.44	0.02
83.	Atypical meningioma	II	61–70	1.25	0.09		
84.	Atypical meningioma	II	71–80	1.43	0.06	1.35	0.01
85.	Anaplastic meningioma	III	41–50	1.42	0.06	1.26	0.03
86.	Anaplastic meningioma	III	41–50	0.86	0.04		
87.	Anaplastic meningioma	III	71–80	0.99	0.04	0.95	0.04
88.	Anaplastic meningioma	III	71–80	1.10	0.04		
89.	Transitional meningioma	I	31–40	1.56	0.08		
90.	Transitional meningioma	I	41–50	1.55	0.09		
91.	Transitional meningioma	I	41–50	1.66	0.08		
92.	Transitional meningioma	I	51–60	1.58	0.05		
93.	Transitional meningioma	I	51–60	1.65	0.11		
94.	Transitional meningioma, recurrent	I	51–60	1.66	0.14		
95.	Meningothelial, transitional and angiomatous meningioma	I	61–70	1.43	0.1		
96.	Transitional meningioma	I	51–60	1.46	0.09		
97.	Meningothelial and transitional meningioma	II	61–70	1.54	0.09	1.67	0.09
98.	Transitional meningioma, partially psammomatous	I	61–70	1.53	0.06	1.63	0.12
99.	Transitional meningioma	I	71–80	1.48	0.04	1.56	0.1
100.	Meningothelial, metaplastic and psammomatous meningioma	I	71–80	1.46	0.06		

**Fig. 2:**
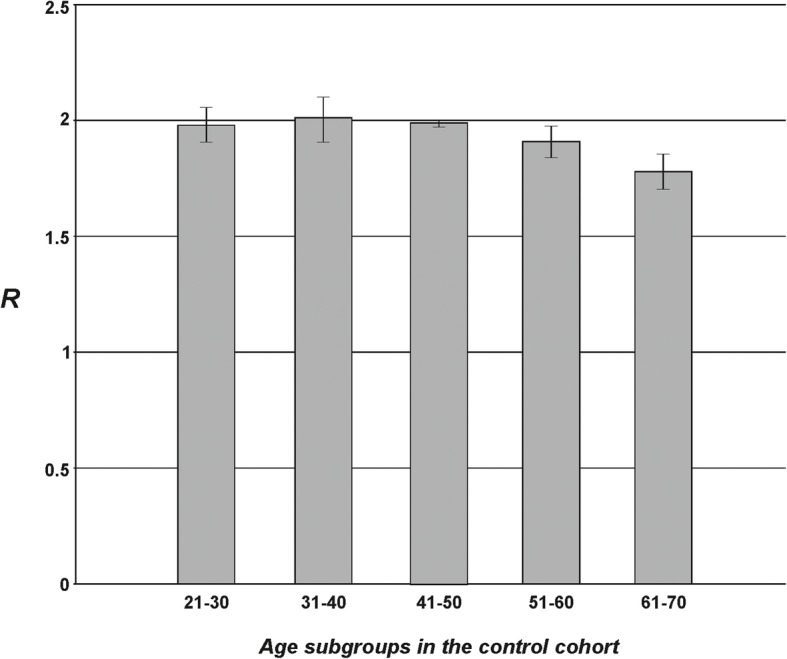
The total m^5^C contents (R) in DNA from peripheral blood samples in the cohort of generally healthy individuals, divided into age subgroups. One can observe that R ca. 2.0 is distinctive for the non-pathologic state

The most abundant histological variant of meningioma in the analyzed cohort was meningothelial (42 cases), followed by fibrous, angiomatous, transitional, mixed, atypical, anaplastic, and psammomatous types (21, 10, 8, 6, 5, 4, and 4 cases respectively). There was no evident predilection of specific tumor variants to patients’ age or sex in the study. Tumor subtypes with the numerical amount and grade distribution are presented in Table 2. The control group consisted of 30 generally healthy persons, aged from 30 to 66 years (mean 49.8 ± 10.5 years), with 11 (36.7%) males and 19 (63.3%) females (Fig. 2).

**Table 2:** Data summary of total DNA methylation in various intracranial meningioma subtypes with numerical amount and grade distribution in all patients evaluated in the study. Median age (in years) in the histological subclasses, and female to male (F:M) ratio is also given

Histological type	Grade	No of cases	Median age [Y]	F:M ratio	R tissue	SD R tissue
Meningothelial meningioma	I	40	54.9	26:14	1.59	0.006
	II	2	53.0	1:1	1.37	0.34
Fibrous meningioma	I	21	60.5	16:5	1.55	0.05
Transitional meningioma	I	8	51.5	7:1	1.58	0.08
Psammomatous meningioma	I	4	60.3	4:0	1.54	0.06
Angiomatous meningioma	I	10	60.9	7:3	1.50	0.03
Mixed meningioma	I	4	62.75	1:3	1.49	0.05
	II	2	64.0	2:0	1.48	0.09
Atypical meningioma	II	5	58.2	3:2	1.45	0.12
Anaplastic meningioma	III	4	60.0	3:1	1.09	0.24

### Global DNA methylation in tumor tissue samples

**Fig. 3:**
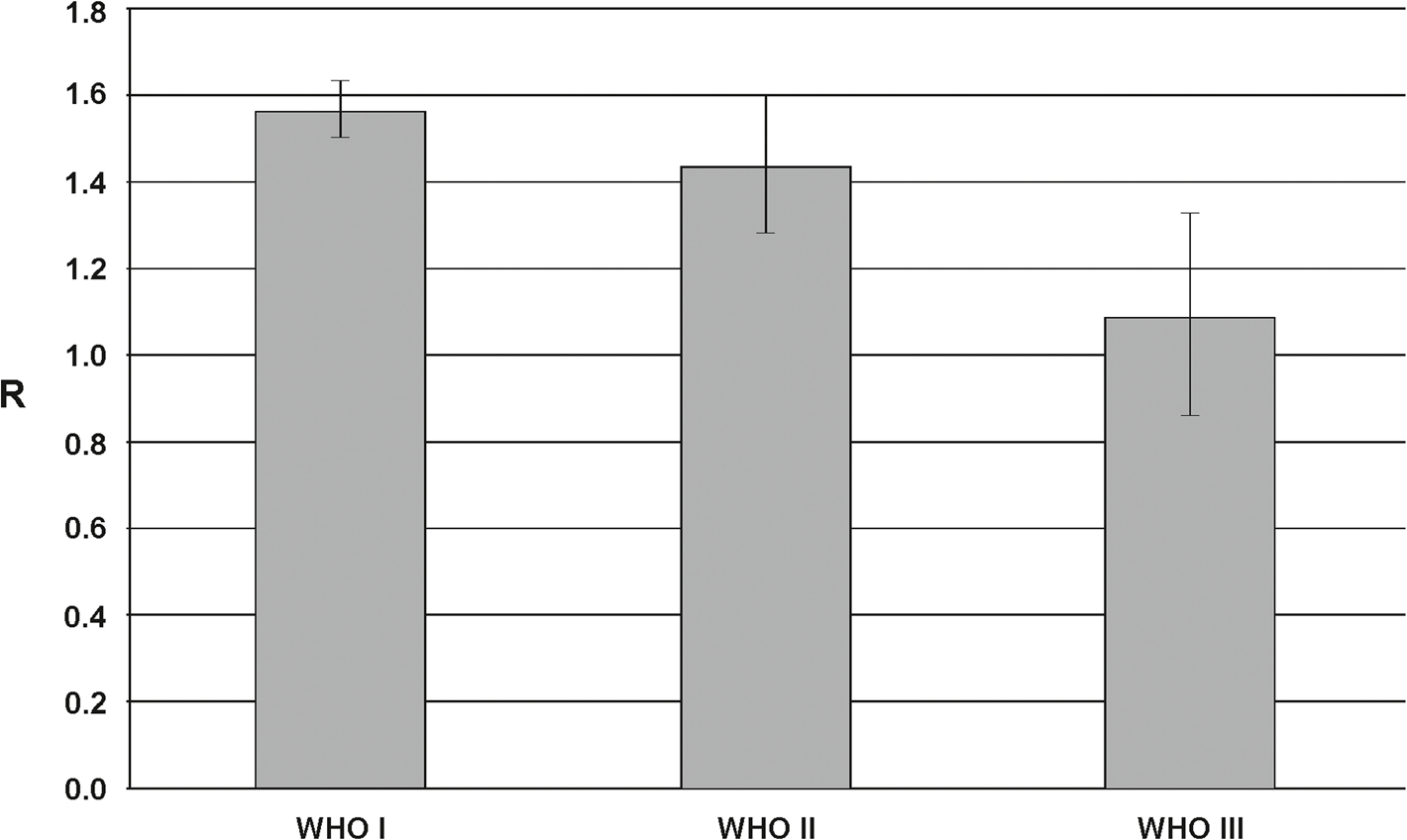
The total m^5^C contents (R) in DNA isolated from different intracranial meningioma tissues divided into the malignancy grades (WHO I represents the tumors of lowest malignancy, while WHO III relates to the most malignant tumors). Grade I tumors are characterized by mean R around 1.6, grade II – 1.4, and grade III – 1.1

For all 100 patients, the total m^5^C amount in genomic DNA extracted from meningioma tumor tissue was analyzed (Tables 1 and 2). The total m^5^C amount expressed as R factor (see Materials and Methods), varies clearly between the patients (Table 1, Supplementary Figure 1), and the groups divided by the histological type (Table 2). The m^5^C level in intracranial meningioma DNA negatively correlates with tumor grade (Fig. 3). The less malignant tumors show higher m^5^C contents than more malignant, and the difference is statistically significant (*F* = 61.796 and *p* < 0.001). No correlation of total DNA methylation (R) was found with patients’ age (*r* = − 0.13) and sex (*r* = 0.21), as well as with specific meningioma subtypes.

### Global DNA methylation in peripheral blood samples

**Fig. 4:**
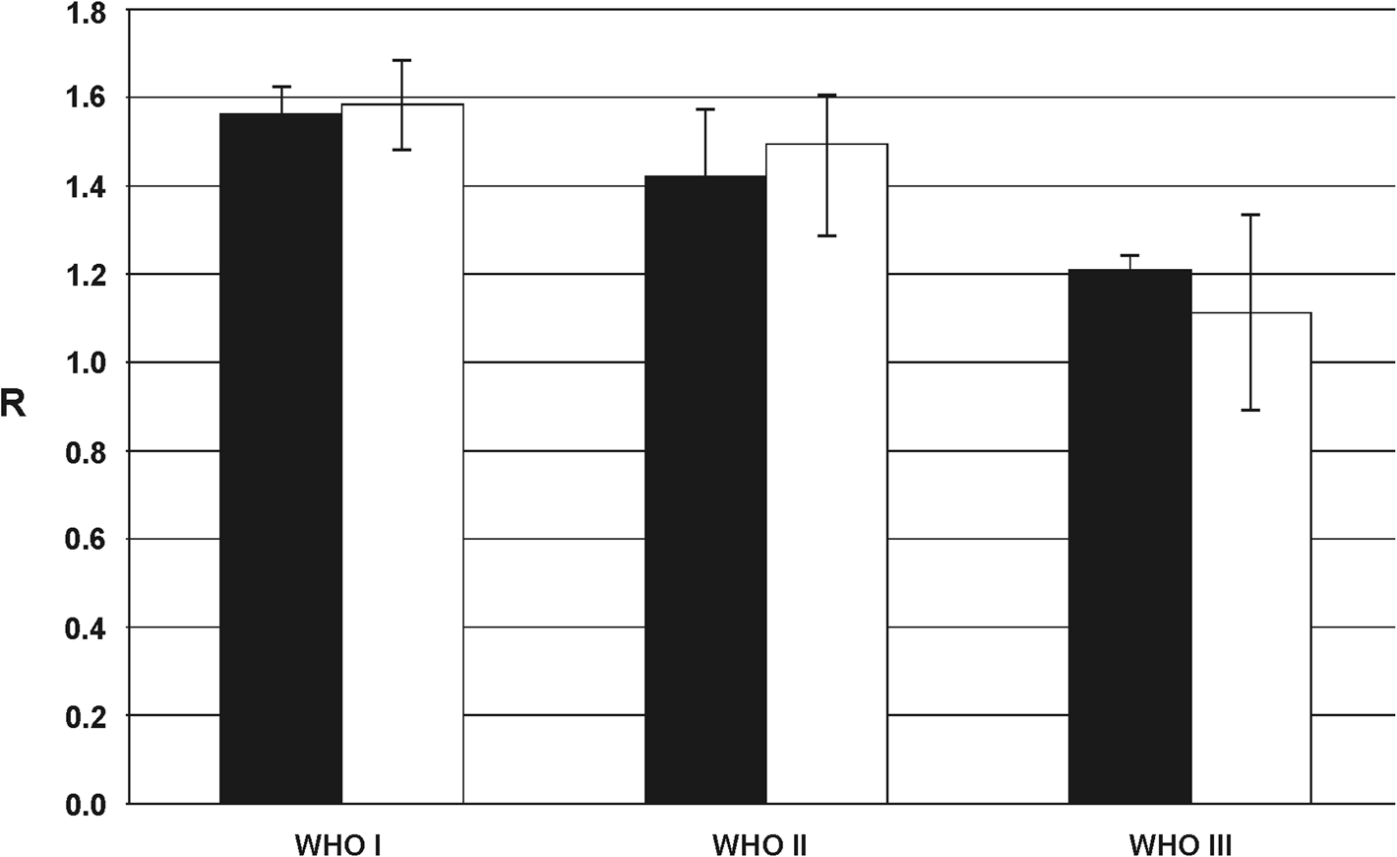
Total amounts of m^5^C in DNA (R) from tumor (black bars) and peripheral blood (white bars) samples of the same subjects with intracranial meningiomas. Total DNA methylation level in liquid biopsy (blood) samples reflect that in tumor tissues allowing noninvasive diagnostics and monitoring

For 29 patients, we also analyzed the total amount of m^5^C in genomic DNA from peripheral blood samples (Tables 1 and 3). The amount of m^5^C expressed as R coefficient varies clearly between the patients (Table 1, Supplementary Figure 2), and the groups divided by the histological type (Table 3). The level of m^5^C in DNA from peripheral blood samples negatively correlates with tumor grade (Fig. 4). Patients with more malignant tumors show lower total m^5^C contents in DNA from peripheral blood samples than less malignant, and the difference is statistically significant (*F* = 18.024 and *p* < 0.001). No correlation of total DNA methylation (R) was found with patients’ age (*r* = 0.34) and sex (*r* = 0.19), as well as with specific meningioma subtypes.

**Table 3:** Data summary of total DNA methylation in tumor tissue and peripheral blood samples of 29 patients with various meningioma types with numerical amount and grade distribution. Median age (in years) in the histological subclasses, and female to male (F:M) ratio is also given

Histological type	Grade	No of cases	Median age	F:M ratio	R tissue	SD R tissue	R blood	SD R blood
Meningothelial meningioma	I	10	53.0	9:1	1.58	0.03	1.60	0.09
	II	1	57.0	1:0	1.13	0.04	1.48	0.08
Fibrous meningioma	I	4	63.75	2:2	1.61	0.07	1.63	0.08
Transitional meningioma	I	1	71.0	1:0	1.48	0.04	1.56	0.1
Angiomatous meningioma	I	5	61.0	3:2	1.50	0.02	1.50	0.12
Mixed meningioma	I	1	69.0	1:0	1.53	0.06	1.63	0.12
	II	2	64.0	2:0	1.48	0.09	1.62	0.07
Atypical meningioma	II	3	65.3	1:2	1.47	0.05	1.41	0.06
Anaplastic meningioma	III	2	58.0	2:0	1.21	0.03	1.11	0.22

In the control group of generally healthy individuals, the mean R coefficient was 1.93 ± 0.10, so significantly higher than in any of the brain meningioma groups (Fig. 2). The ANOVA test for blood results in cancer patients and the control group showed *F* = 90.203 and *p* < 0.001.

### Comparison of total m^5^C contents in genomic DNA from tumor and peripheral blood samples

**Fig. 5:**
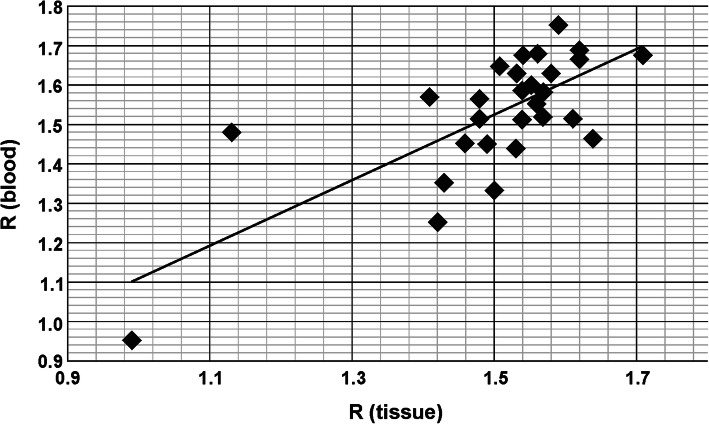
Comparison of total genomic m^5^C contents in DNA from intracranial meningioma tissue and peripheral blood samples of the same subjects. Pearson r correlation factor is 0.72

Total contents of m^5^C in DNA from the intracranial meningioma and peripheral blood samples of the same patients were comparable. The one-way ANOVA test values were: *F* = 0.307 and *p* = 0.581, showing no statistically significant differences between the groups. The relations between the WHO malignancy groups analyzing mean values for global DNA methylation in tumor tissue and blood for the same patients are presented in Fig. 4. The calculated r correlation coefficient for the whole group of patients with the tissue-blood pair was 0.72 (Fig. 5).

## Discussion

While meningiomas are in general benign tumors, difficulties in their management can arise because of surgical limitations, or from aggressive and invasive tumor characteristics, that need adjuvant treatment and radiotherapy [5]. Currently used WHO classification is very subjective and miss reliable markers identifying these recurrence-prone tumors [34]. Recent genome- and exome-wide sequencing approaches have described the meningiomas’ mutational landscape comprehensively defining potential drivers of malignant progression and suggesting potential therapeutic targets [35, 36]. Moreover, the higher rate of genomic disruption was observed among the grade II-III meningiomas than in grade I counterparts (higher rate of non-synonymous mutations and the proportion of the genome affected by somatic copy number alterations) [35].

The established meningioma risk factors (ionizing radiation, head trauma, hormone-replacement therapy, hypertension, and advanced age [37, 38] are all extrinsic and intrinsic stress-inducing factors that can result in oxidative damage of cellular components, also DNA. ROS-induced damage comprises an assembly of DNA lesions that includes base damage, single-strand breaks, and double-strand breaks [39]. Exposure of an organism to chronic stress can result in imbalances of tissue homeostasis and possible tumor formation [40].

Despite its totally low abundance in DNA (4–5% of the total cytosine) of mammalian cells, 5-methylcytosine can be oxidatively damaged and induce mutations. It results in GC → AT transitions at CpG dinucleotides on the genetic level. However, at the epigenetic level, its damage influences the transcription regulation, embryonic development, and other life processes, as well as carcinogenesis [41, 42]. Data are suggesting that global DNA hypomethylation, observed in cancer, increases the instability of the genome. Promoter hypermethylation results in the silencing of genes involved in DNA repair, regulation of cell cycle, initiation of apoptosis, and tumor signaling networks control. All those pathways reflect the hallmarks of cancer [43]. Most frequently, hypermethylated genes encode transcription factors taking part in development, and their methylation may cause a permanent silencing of a gene [44]. Our approach focuses on the estimation of the total amount of m^5^C, regardless of its place in the genome. Through that, we show total DNA damage that is happening on epigenetic (regulatory level) because the epigenetic changes occur earlier and more frequently than genetic ones [45]. Moreover, we’ve recently shown that a decrease in total DNA methylation is a marker of an increase of oxidative damage to the cell, a well-known carcinogenesis factor [24].

Using llumina Infinium HumanMethylation450 BeadChip in genomic-scale DNA methylation profiling Gao et al. identified significantly lower levels of methylation in DNA for malignant meningioma (WHO III) than for atypical (WHO II) or benign (WHO I) ones [29]. Further analysis of different gene regions showed that DNA hypomethylation occurs rather throughout the genome than is restricted to specific gene regions. Therefore, global DNA hypomethylation is observed in the malignant transformation of meningiomas. Changes in global DNA methylation cause gene expression deregulation. Promoter hypermethylation at CpG islands induced gene expression suppression in low and high grade meningiomas, which suggests that DNA methylation is a primary gene silencing mechanism in malignant meningiomas [29].

On the contrary, Harmanci et al. showed a statistically significant positive correlation between the degree of chromosomal alterations and the amount of genome-wide DNA hypermethylation [37]. *NF2* mutant atypical meningiomas displayed a hypermethylated phenotype. There was enrichment in methylation of Polycomb Repressive Complex 2 and Homeobox sites across benign meningiomas compared with control meninges, albeit less than that observed in atypical samples [37].

One should keep in mind that Infinium HumanMethylation450 is a widely-used tool to perform large-scale DNA methylation profiling. However, that method is focused on protein-coding, cancer-associated, and mitochondrial-related genes. The coverage of total CpG sites (the site of high 5-methylcytosine abundance) is low (only around 2%), which means that some features, such as enhancers, are only barely or not at all covered [46].

A multicenter evaluation of genome-wide DNA methylation patterns in 497 samples from meningiomas revealed six different methylation classes presenting with typical mutational, cytogenetic, and gene expression patterns, as well as with clinical relevance [47]. They identified patients with increased risk of tumor progression in the WHO I group, as well as patients with decreased probability of recurrence within the WHO II group. That meningioma classification, based on genome-wide DNA methylation, captured groups that are more homogenous clinically, therefore is more potent than the WHO classification in tumor recurrence prediction and prognosis estimation [47].

Earlier, Kishida et al. distinguished three meningioma clusters based on the methylation status of 6157 genes in 30 meningioma samples [31]. Clusters 2 and 3 included more males compared with cluster 1. The recurrent cases tended to accumulate in cluster 3 compared with clusters 1 and 2. Clusters 2 and 3 were generally hypermethylated, while cluster 1 showed the low methylator phenotype. The proportion of the WHO grades did not show a significant correlation with cluster formation [31].

The recent work of Nassiri et al. presents the methylome-based algorithm that predicts tumor recurrence more reliably than histologic grading, and independently of established clinical and molecular factors [48].

The results of the study presented in this paper show decreasing total DNA methylation with increasing tumor malignancy (Figs. 3 and 4). The method used (TLC separation of radioactively labeled nucleotides from the whole sample’s DNA) allows the estimation of total m^5^C amount in DNA in relation to the total contents of pyrimidines (Fig. 1). The final results come from straightforward calculations, and no data processing is needed. Our approach gives a numerical result, which is reversely correlated with tumor grade. The more malignant the tumor is, the smaller R-value (expressing total DNA methylation) is observed (Figs. 3 and 4). Grade I tumors are characterized by mean R around 1.6, grade II – 1.4, and grade III – 1.1. That tendency is comparable to our previous results for brain gliomas [33] and metastases [49]. However, the mean total DNA methylation levels (R) in those cases were generally lower compared to meningiomas reflecting the known higher malignancy of gliomas and metastasis.

The ultimate goal of every tumor molecular characterization is the establishment of a feature that could serve as a diagnostic or prognostic marker, as well as a therapeutic target [10]. There is limited access to the central nervous system (CNS) tumor tissue just for diagnostic purposes. Unlike many other tumors, the biopsy of intracranial lesions confers a non-negligible degree of risk related to the procedure. A liquid biopsy would facilitate our ability to follow patients longitudinally following initial diagnosis and treatment. Taking into account the aspect of invasiveness, lumbar puncture for cerebrospinal fluid sampling is also a not optimal solution. Therefore, peripheral blood samples are the most obvious choice. The most important question is why and how the global DNA methylation could be the same in tumor and blood samples. The possible answer lies in the mechanism through which DNA methylation is affected by ROS. Because it is a random and global process, not limited to a certain area or cell type, the disease signs can be found in the whole body, not only in the foci where tumors are localized [24, 50]. So far, only a few studies have evaluated the serum samples in CNS neoplasms in the search for DNA/RNA biomarker, mainly for glioma and brain metastasis [51–53], and only single cases for meningiomas in recent years [54–56]. In our previous study, we have analyzed the profile of aberrant methylation of *MGMT*, *RASSF1A*, *p15INK4B*, and *p14ARF* genes in serum free-circulating DNA and corresponding tumor tissue in a group of CNS cancer patients. The comparison of the results obtained for paired serum and tumor samples (also for meningioma) allowed the conclusion that the global concordance of results between these two sample sources is fairly high [57]. Moreover, we performed earlier total DNA methylation analysis with the TLC method for different brain tumors, breast and colon cancers, as well as arterial hypertension. The R values for tumor and blood DNA were comparable, showing that peripheral blood is an adequate source of DNA for complete methylation analysis, and can reflect the state of tumor tissue [58]. In the present study, the total m^5^C contents in DNA from tumor tissue and peripheral blood samples of the same intracranial meningioma patients were almost identical (Figs. 4 and 5). Therefore, it seems that methylation level of DNA from peripheral blood can be used as a diagnostic tool in neurooncology.

## Conclusions

We showed a reciprocal relation of total m^5^C contents in tumor DNA to its malignancy grade. Therefore total DNA methylation of intracranial meningiomas can serve as a tool for their characteristics and malignancy estimation. Moreover, the correlation between total DNA methylation in tumor tissue and peripheral blood samples from the same patient was observed. That puts a light on the tumor pathogenesis (oxidative stress) but also enables the use of peripheral blood as a sample for detecting the tumor or monitoring of the disease. The identification of biomolecular parameters that could improve meningioma classification may optimize the indications for possible adjuvant therapies and closer follow-up.

## Supplementary information

**Additional file 1: Supplementary Figure 1.** The total m^5^C contents (R) in DNA from meningioma tissues of 100 patients evaluated in this study. The bar graph visualizes heterogeneity among the patients. The patients’ order reflects that in Table 1 (where SD values for each data point are included). Meningioma variant names are accompanied by relevant malignancy grade. Grey bars correspond to WHO I, yellow – WHO II, and red – WHO III.

**Additional file 2: Supplementary Figure 2.** The total m^5^C contents (R) in DNA from peripheral blood samples of 29 meningioma patients evaluated in this study. The bar graph visualizes heterogeneity among the patients. The patients’ order reflects that in Table 1 (where SD values for each data point are included). Meningioma variant names are accompanied by relevant malignancy grade. Grey bars correspond to WHO I, yellow – WHO II, and red – WHO III.

## Data Availability

The data generated or analyzed during this study are included in the published article. Further details are available from the corresponding author upon request.

## Abbreviations

C: Cytosine
CNS: Central nervous system
m^5^C: 5-methylcytosine
ROS: Reactive oxygen species
T: Thymine
TLC: Thin layer chromatography
WHO: World Health Organisation
