# Supplementary figures and images for: Total DNA methylation as a biomarker of DNA damage and tumor malignancy in intracranial meningiomas
Source: BMC Cancer. 2020 Jun 3;20:509. doi: 10.1186/s12885-020-06982-3 (PMC7268775; doi:10.1186/s12885-020-06982-3)

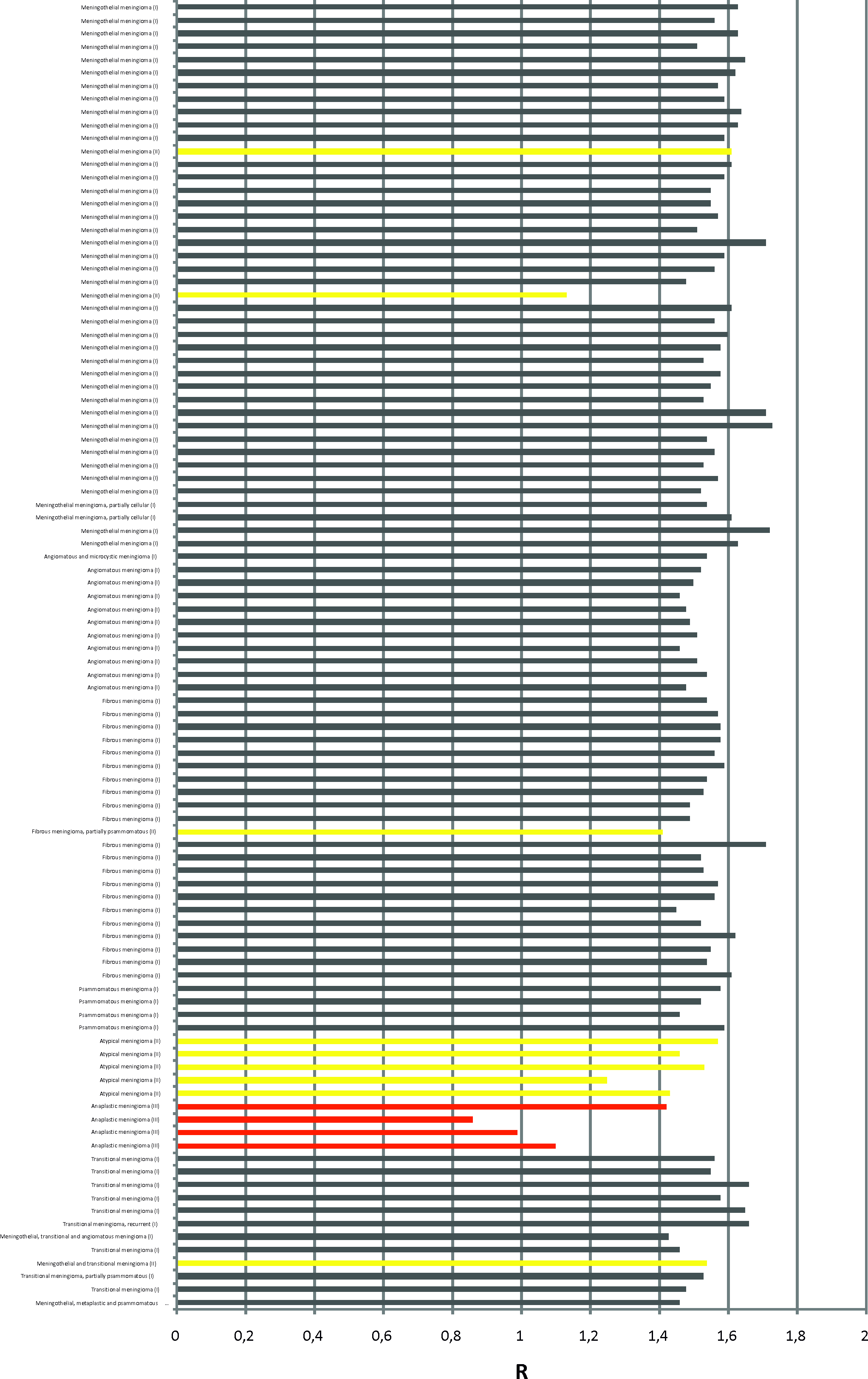

Supplement: Supplementary file 1 — Additional file 1: Supplementary Figure 1. The total m5C contents (R) in DNA from meningioma tissues of 100 patients evaluated in this study. The bar graph visualizes heterogeneity among the patients. The patients’ order reflects that in Table 1 (where SD values for each data point are included). Meningioma variant names are accompanied by relevant malignancy grade. Grey bars correspond to WHO I, yellow – WHO II, and red – WHO III. [file 12885_2020_6982_MOESM1_ESM.tif]

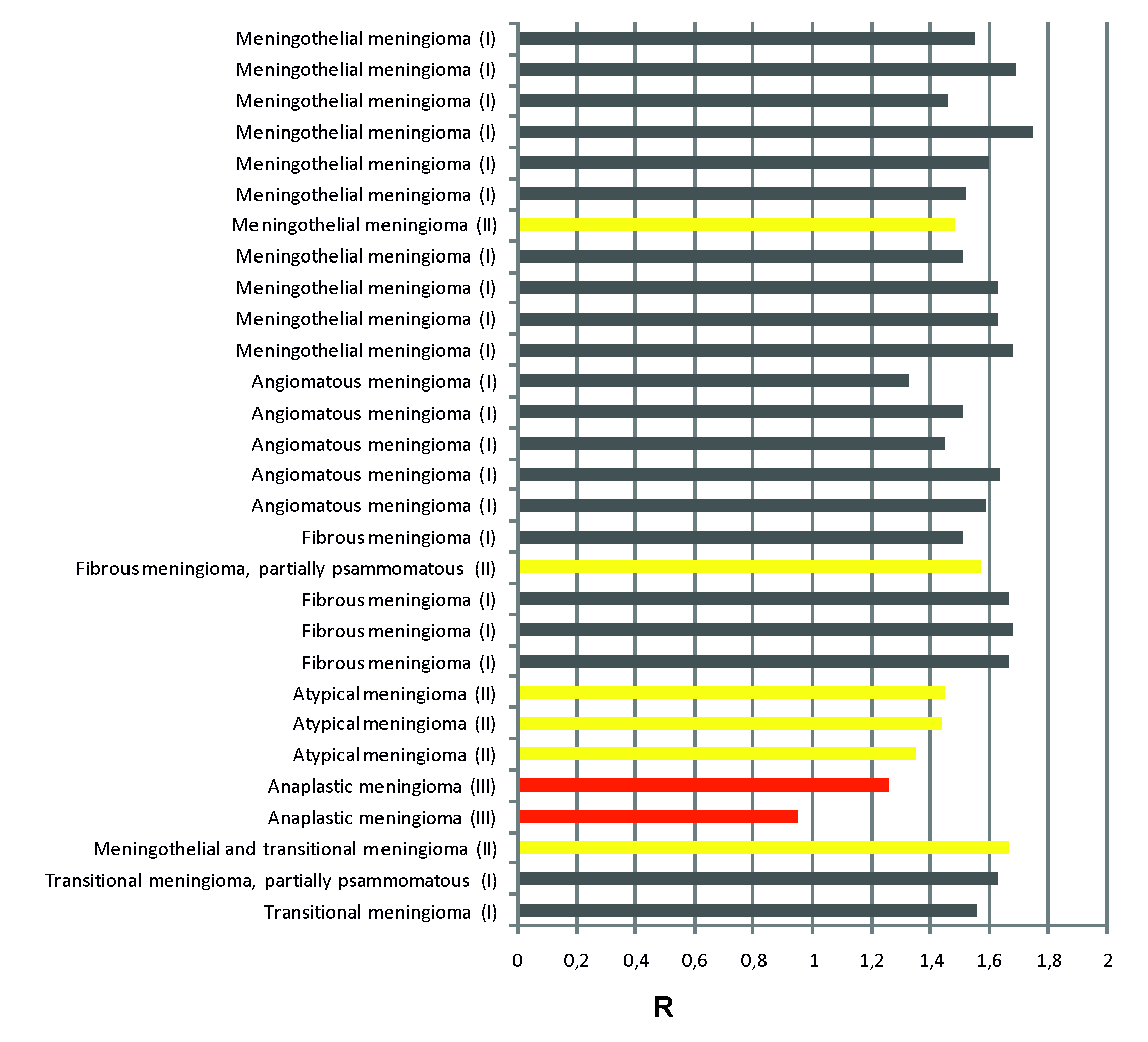

Supplement: Supplementary file 2 — Additional file 2: Supplementary Figure 2. The total m5C contents (R) in DNA from peripheral blood samples of 29 meningioma patients evaluated in this study. The bar graph visualizes heterogeneity among the patients. The patients’ order reflects that in Table 1 (where SD values for each data point are included). Meningioma variant names are accompanied by relevant malignancy grade. Grey bars correspond to WHO I, yellow – WHO II, and red – WHO III. [file 12885_2020_6982_MOESM2_ESM.tif]
